# Valorization of *Zingiber officinale* Roscoe leaf biomass: phytotoxic potential and chemical profiling toward sustainable weed management

**DOI:** 10.1002/jsfa.70840

**Published:** 2026-06-25

**Authors:** Sara Vitalini, Orlando Innaurato, Shadab Faramarzi, Alessandro Palmioli, Stefania Garzoli, Valentina Vaglia, Cristina Airoldi, Marcello Iriti

**Affiliations:** ^1^ One Health Section, Department of Biomedical, Surgical and Dental Sciences Università degli Studi di Milano Milan Italy; ^2^ Department of Horticultural Sciences and Engineering Campus of Agriculture and Natural Resources, Razi University Kermanshah Iran; ^3^ Department of Biotechnology and Biosciences, BioOrgNMR Lab Università degli Studi di Milano – Bicocca Milan Italy; ^4^ Department of Drug Chemistry and Technology Sapienza Università di Roma Rome Italy; ^5^ Department of Earth and Environmental Sciences Università di Pavia Pavia Italy

**Keywords:** agricultural by‐products, allelochemicals, *C*‐glycosyl flavones, circular economy, ginger, volatile terpenoids

## Abstract

**BACKGROUND:**

*Zingiber officinale* Roscoe is widely cultivated as a spice and functional food, generating substantial amounts of aerial biomass that is commonly discarded after harvest. Within a circular‐economy framework, this study investigated the phytotoxic potential of *Z. officinale* leaf biomass and its relevance for sustainable agricultural systems.

**RESULTS:**

Leaf powder and aqueous extracts were evaluated under pre‐ and post‐emergence conditions using selected weed species [*Echinochloa oryzoides* (Ard.) Fritsch, *Lolium multiflorum* Lam., *Sinapis alba* L. and *Trifolium incarnatum* L.] and *Oryza sativa* L. as a reference crop. Both matrices exerted significant inhibitory effects on seed germination and early seedling development, with marked differences in species sensitivity and lower susceptibility of the crop species across filter paper and soil substrates. Post‐emergence assays further confirmed species‐dependent responses. Chemical profiling by solid‐phase microextraction‐gas chromatography/mass spectrometry revealed a volatile fraction dominated by sesquiterpenes, mainly *β*‐caryophyllene, whereas ultra‐performance liquid chromatography‐high‐resolution mass spectrometry and NMR analyses identified *C*‐glycosylated flavones, primarily apigenin derivatives, in the aqueous extract.

**CONCLUSION:**

These findings highlight *Z. officinale* leaves as a promising biologically active by‐product for low‐impact weed management strategies and agricultural residue valorization. © 2026 The Author(s). *Journal of the Science of Food and Agriculture* published by John Wiley & Sons Ltd on behalf of Society of Chemical Industry.

## INTRODUCTION

The circular economy aims to address the growing scarcity of raw materials and the need for effective waste management by promoting more resilient systems for society and the environment through responsible practices and innovative solutions that support food supply and ecological security.^1^, ^2^

In agriculture, these principles are already embedded in practices such as crop management, where organic amendments such as compost and plant extracts are employed to enhance productivity.^3^, ^4^ Among the main challenges, weeds remain a major constraint, as they compete with crops for essential resources and act as reservoirs for pests, leading to yield losses ranging from approximately 5% in industrialized countries to 10–25% in developing regions.^5^ The widespread adoption of synthetic herbicides to mitigate these impacts has resulted in the emergence of herbicide‐resistant weeds, with 541 current cases involving 273 species (156 dicots and 117 monocots),^6^ at the same time as raising environmental and health concerns that call for sustainable management strategies.

In this context, natural phytotoxic compounds represent a promising alternative. In particular, allelochemicals released by plants can influence the germination and growth of neighboring species, offering opportunities for the development of low‐impact approaches to weed control.^7^, ^8^, ^9^

Agricultural residues from organic production systems represent a valuable yet largely underutilized resource for environmentally compatible agricultural inputs. Among crops attracting increasing attention as a result of their economic relevance and diversified applications, *Zingiber officinale* Roscoe (Zingiberaceae) has experienced a marked expansion in cultivation driven by growing consumer demand.^10^ As a spice and functional food crop, its processing generates residues that constitute agri‐food by‐products. After rhizome harvesting, considerable quantities of aerial biomass are typically discarded despite their potential as a source of biologically active secondary metabolites.

Although the chemical composition and biological properties of *Z. officinale* rhizomes are well documented, the phytotoxic or allelopathic potential of this species has received relatively limited attention. Existing evidence is largely restricted to biological assays using cultivated species as target plants, often lacking chemical characterization or comparative evaluation among different plant‐derived preparations.^11^, ^12^, ^13^ More recent metabolomic studies have focused primarily on rhizome extracts and isolated constituents.^14^

Evaluating phytotoxic responses across taxonomically and functionally distinct receiver species, including problematic agricultural weeds and crop species, may provide useful information on selectivity and potential applicability in sustainable weed‐management systems.^15^ In particular, weeds associated with herbicide‐resistant biotypes represent relevant targets for the investigation of alternative plant‐derived bioactive materials and bioherbicidal strategies.^6^, ^15^

In the present study, the phytotoxic effects of *Z. officinale* leaf biomass were investigated by combining pre‐ and post‐emergence bioassays on selected weed species with *Oryza sativa* L. as a reference crop. Biological responses were interpreted in light of complementary chemical analyses, providing an integrated assessment of their inhibitory activity and highlighting their potential relevance as an agricultural by‐product.

## MATERIALS AND METHODS

### Plant material

*Z. officinale*, used as the donor species, was supplied by the ‘Angelo Salera’ farm (Cividate al Piano, Bergamo province, northern Italy), where it was organically grown under greenhouse conditions. The plants were harvested in November 2019. After separation from the rhizomes, aerial parts were collected, and the leaves were dried in a ventilated oven at 40 °C, ground to powder, and stored under dry conditions until use for bioassays and extraction procedures.

The species identity was verified based on morphological characteristics and commercial traceability data provided by the supplier, in accordance with World Flora Online.^16^ A voucher specimen (no. ZO‐0320‐SVFDC) was deposited at the One Health Section, Department of Biomedical, Surgical and Dental Sciences, University of Milan.

Seeds of the receiver monocots *Echinochloa oryzoides* (Ard.) Fritsch, *Lolium multiflorum* Lam., and *O. sativa* L. were provided by the organic farm ‘Terre di Lomellina’ (Italy), whereas those of the dicots *Sinapis alba* L. and *Trifolium incarnatum* L. were purchased from ‘Padana Sementi’ (Padova, Italy). These species were selected based on agronomic relevance and functional diversity, including weeds associated with agricultural systems and differing in ecological and physiological traits. The selected panel also included species for which herbicide‐resistant biotypes have been reported, together with *O. sativa* as a reference crop species. All seed lots were stored at 4 °C upon receipt and surface‐sterilized in 1% (v/v) sodium hypochlorite for 10 min before use.

### Solid–liquid extraction

Aqueous extracts were obtained by maceration of *Z. officinale* leaf powder in distilled water (1:10, w/v) under continuous stirring at room temperature for 24 h. The suspension was filtered through gauze and centrifuged at 3500 rpm (2180 × g) for 30 min. The resulting supernatant was used either undiluted or diluted to 50%.^17^

### Solid‐phase microextraction (SPME)

Volatile compounds from leaf powder (0.5 g) and aqueous extract (1 mL) were sampled by SPME using a DVB/CAR/PDMS (i.e. divinylbenzene/carboxen/polydimethylsiloxane) fiber (50/30 μm). Samples were equilibrated at 50 °C for 20 min, followed by fiber exposure for 45 min at the same temperature.

Desorption was performed in the gas chromatography (GC) injector at 250 °C.

### Gas chromatography/mass spectrometry (GC/MS) analysis

Volatile profiles were analyzed using a GC‐MS system (Clarus 500; PerkinElmer, Shelton, CT, USA) equipped with a VF‐1 capillary column (Agilent, Santa Clara, CA, USA). The oven temperature was programmed from 60 °C to 220 °C at 6 °C min^−1^. Mass spectra were acquired in electron impact mode (70 eV). Compound identification was based on comparison with Wiley (https://sciencesolutions.wiley.com/mass-spectral-databases) and NIST (https://www.nist.gov/) libraries and linear retention indices calculated using a C_8_–C_30_ *n*‐alkane series. Relative abundances were expressed as peak area percentages. Analyses were performed in triplicate. Analytical conditions were adapted from previously published SPME‐GC/MS protocols successfully applied to the volatile profiling of complex plant matrices.^18^, ^19^

### Ultra‐performance liquid chromatography/electrospray ionization‐high‐resolution mass spectrometry (UPLC/ESI‐HR‐MS) analysis

UPLC/ESI‐HR‐MS analyses were performed using a Waters® Acquity™ UPLC system coupled to a Xevo G2‐XS QTof mass spectrometer equipped with an ESI source and controlled by MassLynx™ 4.2 software (Waters, Milford, MA, USA). The aqueous extract was diluted five‐fold in water/acetonitrile (90:10, v/v) and 2 μL was injected. Separation was achieved on a Waters® Acquity™ Premier HSS T3 column (100 × 2.1 mm inner diameter, 1.8 μm) with a corresponding guard column.

The mobile phase consisted of MS‐grade water (A) and acetonitrile (B), both containing 0.1% (v/v) formic acid, at a flow rate of 0.4 mL min^−1^ and a column temperature of 40 °C. Elution was performed using the gradient: 5% B (0–1 min), linear increase to 50% B (1–11 min), 90% B (11–12 min), column washing at 90% B (12–15 min), return to initial conditions (15–16 min) and re‐equilibration for 4 min.

Mass spectra were acquired in both positive and negative ion modes using data‐independent acquisition [mass spectrometry elevated energy (MS^E^)]. Source parameters were set as: capillary voltage, +3/−2 kV; cone voltage, 40 V; source temperature, 120 °C; desolvation temperature, 350 °C; desolvation gas flow, 1000 L h^−1^; and cone gas flow, 50 L h^−1^. Data were collected over the range *m*/*z* 50–1200. Mass calibration was performed using sodium formate, with leucine enkephalin used as lock mass.

Analytical conditions and data‐processing workflow were adapted from previously published UPLC/ESI‐HR‐MS methodologies applied to natural matrices.^20^, ^21^, ^22^

### Nuclear Magnetic Resonance (NMR) spectroscopy

An aliquot of the aqueous extract was freeze‐dried and resuspended in deuterated phosphate buffer (20 mm) to a final concentration of 30 mg mL^−1^, containing d9‐trimethylsilylpropionic acid (TSP, 1mM) as internal standard for chemical shift referencing and quantification. The pH was adjusted to 7.4 using NaOD or DCl, accounting for isotope effects.

NMR spectra were acquired at 298 K on an Avance III 600 MHz spectrometer equipped with a QCI cryoprobe (Bruker, Billerica, MA, USA). One‐dimensional ^1^H NMR spectra were recorded using nuclear Overhauser effect spectroscopy‐presat, Carr‐Purcell‐Meiboom‐Gill‐presat and LED pulse sequences with 256 scans, a spectral width of 20 ppm and a relaxation delay of 5 s. Spectra were processed with 0.3‐Hz line broadening, automatic phase and baseline correction, and calibrated to the TSP signal at 0.0 ppm.

Two‐dimensional ^1^H—^1^H TOCSY and ^1^H—^13^C HSQC experiments were acquired to support metabolite identification. Data were processed using MestReNova, version 14.3.0 (https://mestrelab.com). Metabolite identification and assignment were performed by comparison with internal databases and published data, and compound concentrations were estimated from multiple assigned signals when possible, in accordance with previously described protocols.^23^, ^24^, ^25^, ^26^, ^27^ Concentrations were expressed as mg L^−1^ in the initial aqueous extract.

### Pre‐emergence phytotoxicity assay

Pre‐emergence phytotoxicity was evaluated under controlled laboratory conditions using *Z. officinale* leaf powder and its aqueous extracts in separate assays, in accordance with previously described protocols.^28^

Fifteen seeds of each receiver species were placed either on a filter paper disk or sown in 25 g of sterilized topsoil (SER CA‐V7; Vigorplant, Fombio, Italy) in 90‐mm Petri dishes under a sterile vertical laminar airflow hood.

For powder assays, filter paper and soil substrates were moistened with 5 and 15 mL of distilled water, respectively. Leaf powder was applied at 0.25 g per Petri dish on filter paper or mixed into soil at three concentrations (1:100, 1:50 and 1:25, w/w). Control samples received distilled water only, without addition of leaf powder.

For extract assays, filter paper and soil substrates received 5 and 15 mL of aqueous extract, respectively, applied at both 50% and 100% concentrations. Control samples received distilled water only.

Petri dishes were sealed with Parafilm® (Amcor, Zurch, Switzerland) and incubated in a climatic chamber at 25 °C (16 h of light) and 18 °C (8 h of dark). The experimental design included five receiver species, two to four treatment concentrations (including controls), three replicates per condition and two independent runs. Under pre‐emergence conditions, seed germination and early seedling development occur simultaneously and were therefore evaluated together as indicators of overall phytotoxic pressure.

### Post‐emergence phytotoxicity assay

Post‐emergence phytotoxicity was assessed on seedlings of the five receiver species. Seeds were germinated in Petri dishes under controlled conditions (25 °C, 16 h of light/18 °C, 8 h of dark) and subsequently transferred to pots (15 seedlings per pot, diameter 13 cm) containing SER CA‐V7 (Vigorplant) topsoil. Three weeks after transplantation, when the first leaves were fully expanded, seedlings were treated with undiluted *Z. officinale* aqueous extract applied as a foliar spray to the point of runoff. Control plants were treated with distilled water only. Phytotoxic symptoms were recorded 48 h after treatment. Each treatment included six replicates and was repeated in two independent experimental runs.

### Assessment of germination and growth parameters

Seed germination was monitored daily for 7 days. At the end of the experiment, root and shoot lengths of seedlings grown on filter paper and shoot emergence from soil were measured. These parameters were considered integrative descriptors of early seedling performance under pre‐emergence conditions.

Germination and growth responses were quantified using the following indices: germination percentage (*G*), coefficient of velocity of germination (CVG), mean germination time (MGT) and seedling vigour index (SVI).^29^, ^30^, ^31^

Post‐emergence phytotoxic effects were assessed using a semi‐quantitative visual injury scale adapted by Robles *et al*.^32^ and Nalini and Parthasarathi.^33^ The scale classified plant damage into ten severity classes based on the percentage of injured tissues and associated symptoms (Table 1).

**Table 1:** Semi‐quantitative scale used for the assessment of post‐emergence phytotoxic injury induced by *Zingiber officinale* aqueous extract on receiver species seedlings

Class	% injury	Core symptoms
I	1–10	Very slight discoloration (mild yellowing or browning)
II	11–20	Slight discoloration, distortion, stunting
III	21–30	Moderate discoloration, stunting and twisting (no burning)
IV	31–40	Severe twisting with up to 40% leaf burning, possible permanent damage
V	41–50	Visible damage affecting up to 50% of plant tissues, leaves burnt or lost
VI	51–60	Severe damage affecting more than 50% of leaves burnt or lost
VII	61–70	Severe damage affecting more than 60% of plant tissues
VIII	71–80	Very severe damage, tissues nearly destroyed (about 75%)
IX	81–90	Almost complete necrosis; collapse of stand or 90% leaves burnt/lost
X	91–100	Complete destruction: 100% tissue necrosis/plant death

### Statistical analysis

Data were analyzed using SPSS (IBM Corp., Armonk, NY, USA). Prior to analysis, data were checked for normality and homogeneity of variances. For pre‐emergence assays, independent‐samples *t*‐tests were applied to datasets including two treatment groups, whereas one‐way analysis of variance (ANOVA) followed by Tukey's post‐hoc test (*P* < 0.05) was applied to experiments including more than two treatment levels, separately for each substrate and treatment condition, to evaluate effects on germination and growth indices.

A two‐way ANOVA was additionally performed considering treatment type (powder or extract) and species as fixed factors to assess interaction effects and species‐specific responses, including differences between grass and broadleaf species.

For post‐emergence assays, phytotoxicity data expressed as cumulative injury percentages were analyzed by one‐way ANOVA followed by Tukey's test (*P* < 0.05).

## RESULTS

### SPME‐GC/MS profiling of *Z. officinale* powder and extract

The results showed that the volatile fraction of *Z. officinale* was dominated by sesquiterpene hydrocarbons in both the powder (84.2%) and the aqueous extract (90.5%), although the two matrices exhibited clear compositional differences (Table 2). In the powder, *β*‐caryophyllene was the most abundant constituent (54.8%), followed by *γ*‐muurolene (19.2%), *α*‐farnesene (3.9%) and *α*‐copaene (2.5%). Oxygenated sesquiterpenes accounted for 7.4% of the total fraction, mainly represented by caryophyllene oxide (5.0%). Monoterpenes accounted for 8.4%, with *α* ‐curcumene (5.8%) as the main compound. In the aqueous extract, *β*‐caryophyllene reached 90.5% of the total, whereas caryophyllene oxide was the only oxygenated sesquiterpene detected (1.3%). The monoterpene fraction (8.2%) displayed a different distribution compared with the powder, with *β*‐pinene (7.3%) becoming the predominant component.

**Table 2:** Volatile composition of *Zingiber officinale* leaf powder and aqueous extract analyzed by SPME‐GC/MS. Relative abundances are expressed as percentage mean values ± SD (*n* = 3)

Components	LRI^a^	LRI^b^	Powder (%), mean ± SD	Extract (%), mean ± SD
**Monoterpene hydrocarbons**				
*α*‐Pinene	938	941	1.0 ± 0.01	0.7 ± 0.03
*β*‐Pinene	975	978	0.9 ± 0.03	7.3 ± 0.13
*β‐*Τhujene	966	968	0.7 ± 0.02	0.2 ± 0.02
*α*‐Curcumene	1468	1475	5.8 ± 0.12	–
**Sesquiterpene hydrocarbons**				
*α*‐Copaene	1381	1379	2.5 ± 0.10	–
*β*‐Caryophyllene	1428	1431	54.8 ± 2.15	90.5 ± 5.60
Aromadendrene	1455	1460	1.6 ± 0.08	–
*α*‐Farnesene	1478	1482	3.9 ± 0.07	–
*γ*‐Muurolene	1505	1501	19.2 ± 0.20	
*δ*‐Cadinene	1522	1518	2.2 ± 0.06	–
**Oxygenated sesquiterpenes**				
*β*‐Eudesmol	1660	1659	2.4 ± 0.07	–
Caryophyllene oxide	1578	1580	5.0 ± 0.09	1.3 ± 0.06
TOTAL			100.0	100.0
Monoterpene hydrocarbons			8.4	8.2
Sesquiterpene hydrocarbons			84.2	90.5
Oxygenated sesquiterpenes			7.4	1.3

^a^ Linear retention indices (LRI) determined on an apolar column.

^b^ Linear retention indices from the literature. Values are expressed as the mean ± SD of three independent analyses. –, Not detected. ‘TOTAL’ indicates the sum of identified compounds within each chemical class.

### UPLC/ESI‐HR‐MS and NMR profiling of *Z. officinale* extract

**Figure 1:**
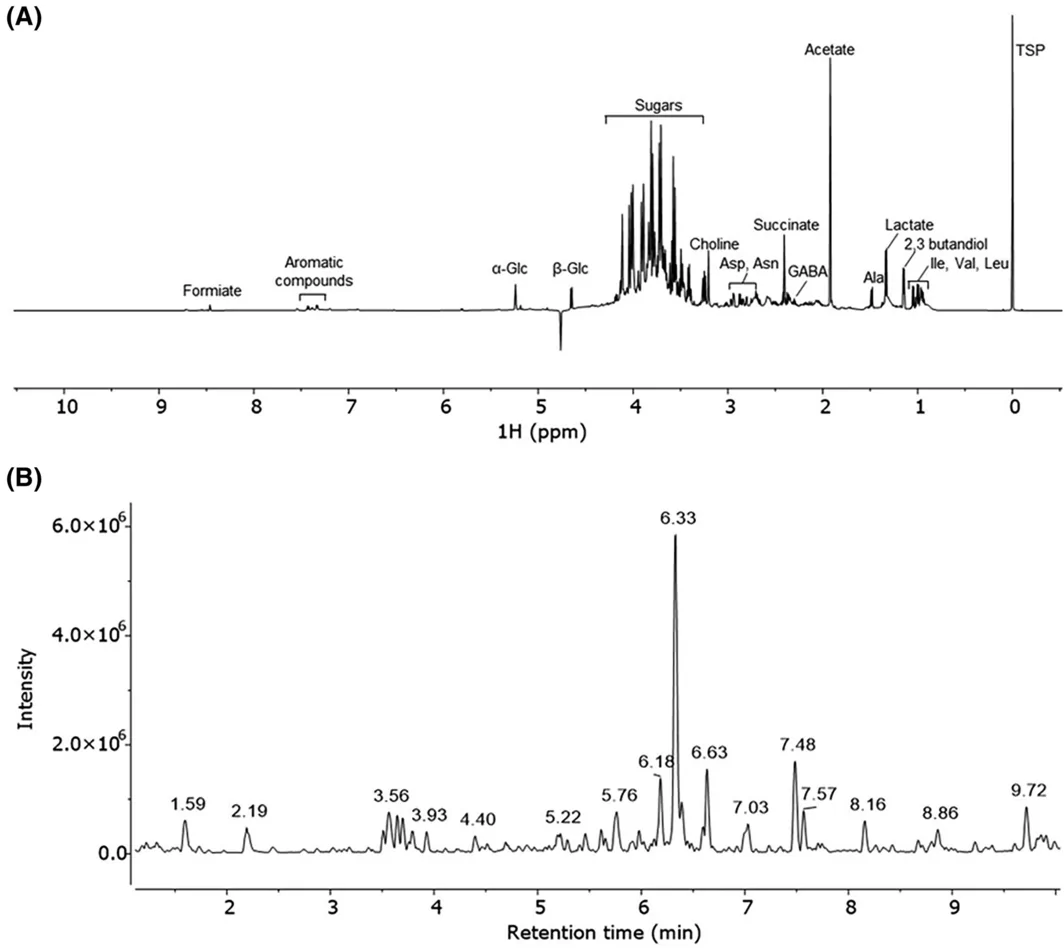
(A) ^1^H‐NMR profile of freeze‐dried *Zingiber officinale* leaf extract dissolved in d‐PB (20 mm, pH 7.2) at a concentration of 30 mg mL^−1^, acquired at 600 MHz and 300 K. Major spectral regions and representative metabolites are indicated. *α*‐Glc and *β*‐Glc refer to the anomeric protons of *α*‐ and *β*‐glucose, respectively. Ala, alanine; Asp, aspartate; Asn, asparagine; GABA, *γ*‐aminobutyric acid; Ile, isoleucine; Leu, leucine; Val, valine; TSP, trimethylsilylpropionic acid, used as internal standard for chemical shift referencing (0.00 ppm). (B) Base peak chromatogram obtained by UPLC‐ESI‐HR‐MS analysis of the *Z. officinale* leaf aqueous extract in negative ionization mode. Peak assignments corresponding to the indicated retention times are reported in Table 3.

The chemical profile of the *Z. officinale* aqueous extract was investigated by combining NMR spectroscopy and UPLC/ESI‐HR‐MS. NMR spectroscopy was employed for comprehensive metabolite identification and quantification, whereas UPLC/HR‐MS was used complementarily to characterize secondary metabolites, particularly polyphenols, through an untargeted approach based on a data‐independent acquisition (MS^E^) in both positive and negative ionization modes. Figure 1(A) shows the ^1^H‐NMR spectrum and Figure 1(B) shows the base peak chromatogram of the *Z. officinale* extract. NMR data analysis revealed the presence of several primary metabolites, including glucose, amino acids and organic acids. NMR peaks assignments and metabolite quantification are reported in Table 3. UPLC/ESI‐HR‐MS analysis revealed the presence of several *C*‐glycoside flavones, mainly sharing apigenin as the aglycone. Identified compounds include apigenin 6,8‐*C*‐diglucoside (vicenin‐2), apigenin 6‐*C*‐arabinoside‐8‐*C*‐glucoside (isoschaftoside), apigenin 6‐*C*‐glucoside‐8‐*C*‐arabinoside (schaftoside), apigenin 6‐*C*‐arabinoside‐8‐*C*‐xyloside and several apigenin 6,8‐*C*‐dipentoside isomers (Table 4).

**Table 3:** ^1^H NMR assignment and quantification of primary metabolites identified in the aqueous extract of *Zingiber officinale*. Concentrations are expressed as mg L^−1^ of extract

Metabolite	Chemical shift (ppm)	Concentration (mg L^−1^)
2,3 Butanediol	1.15 (*d*, *J* = 5.95 Hz), 3.63 (*m*), 3.73 (*m*)	89.93
Acetate	1.92 (*s*)	111.56
Alanine	1.48 (*J* = 7.37 Hz), 3.79 (m)	34.13
Asparagine	2.86 (*dd*, *J* = 16.89, 7.66 Hz), 2.96 (*dd*, *J* = 16.82, 4.04 Hz), 4.02 (*m*)	209.23
Aspartate	2.68 (*dd*, *J* = 17.43, 8.71 Hz), 2.82 (*dd*, *J* = 17.48, 3.72 Hz), 3.90 (*m*)	119.23
Choline	3.21 (*s*)	20.16
Formate	8.46 (s)	6.43
GABA	2.30 (*t*, *J* = 7.52 Hz), 3.02 (*t*, *J* = 7.52 Hz)	36.94
Glucose	4.65 (*d*, *J* = 8.02 Hz, H1β), 5.24 (*d*, *J* = 3.75 Hz, H1α)	493.57
Isoleucine	0.94 (*t*, *J* = 7.34 Hz), 1.01 (*d*, *J* = 7.09 Hz), 1.30 (*m*), 1.48 (*m*), 1.98 (*m*)	36.59
Lactate	1.33 (*d*, *J* = 6.86 Hz), 4.12 (*m*)	91.35
Leucine	0.97 (*m*), 1.72 (*m*), 3.75 (*m*)	58.96
Succinate	2.41 (*s*)	59.30
Valine	1.00 (*d*, *J* = 7.00 Hz), 1.05 (*d*, *J* = 7.04 Hz), 2.29 (*m*), 3.62 (*m*)	48.35

*Note:* ^1^H chemical shifts are reported with multiplicity and coupling constants (J, Hz). Concentrations were estimated from integrated NMR signals using TSP as internal standard and represent single determinations for the analyzed extract.

**Table 4:** Compounds tentatively identified in the aqueous extract of *Zingiber officinale* by UPLC–ESI–HR‐MS using an untargeted approach in negative ion mode

#	RT (min)	Compound	Molecular formula	Monoisotopic mass	Adduct	Experimental (*m*/*z*)	AbsErr (ppm)	InChlKey
1	2.19	l‐Phenylalanine	C9H11NO2	165.0790	[M‐H]^−^	164.0716	0.38	COLNVLDHVKWLRT‐UHFFFAOYSA‐N
2	3.64	Glucosyringic acid	C15H20O10	360.1056	[M‐H]^−^	359.0980	0.97	BLKMDORKRDACEI‐OVKLUEDNSA‐N
3	3.79	l‐Tryptophan	C11H12N2O2	204.0898	[M‐H]^−^	203.0823	1.37	QIVBCDIJIAJPQS‐UHFFFAOYSA‐N
4	4.40	2‐isopropylmalic acid	C7H12O5	176.0685	[M‐H]^−^	175.0607	2.65	BITYXLXUCSKTJS‐UHFFFAOYSA‐N
5	5.29	Apigenin 6,8‐*C*‐diglucoside (Vicenin‐2)	C27H30O15	594.1585	[M‐H]^−^	593.1512	0.09	FIAAVMJLAGNUKW‐UHFFFAOYSA‐N
6	5.46	UNKN	C14H24O10	351.1297	[M‐H]^−^	351.1293	1.04	‐
7	5.60	Apigenin‐6‐*C*‐xyloside‐8‐*C*‐glucoside (Vicenin‐1)	C26H28O14	564.1406	[M‐H]^−^	563.1406	0.01	OVMFOVNOXASTPA‐MCIQUCDDSA‐N
8	5.76	Apigenin‐6‐ arabinoside ‐8‐glucoside (Isoschaftoside)	C26H28O14	564.1406	[M‐H]^−^	563.1403	0.55	OVMFOVNOXASTPA‐VYUBKLCTSA‐N
9	5.91	Apigenin‐6,8‐*C*‐dipentoside isomer	C25H26O13	534.1373	[M‐H]^−^	533.1297	0.68	‐
10	5.98	Apigenin‐6‐glucoside −8‐ arabinoside (Schaftoside)	C26H28O14	564.1406	[M‐H]^−^	563.1398	1.52	MMDUKUSNQNWVET‐UHFFFAOYSA‐N
11	6.03	Robinin	C33H40O19	740.2164	[M‐H]^−^	739.2067	3.27	PEFASEPMJYRQBW‐LUUUVORTNA‐N
12	6.15	Neohesperidin dihydrochalcone	C28H36O15	612.2054	[M‐H]^−^	611.1973	1.34	ITVGXXMINPYUHD‐UHFFFAOYNA‐N
13	6.18	Apigenin‐6,8‐*C*‐di‐pentoside isomer	C25H26O13	534.1373	[M‐H]^−^	533.1294	1.25	‐
14	6.33	Apigenin 6‐*C*‐*α*‐l‐arabinopyranosyl‐8‐*C*‐*β*‐d‐xylopyranoside	C25H26O13	534.1373	[M‐H]^−^	533.1298	0.45	LDVNKZYMYPZDAI‐UHFFFAOYSA‐N
15	6.60	*N*‐Acetyltryptophan	C13H14N2O3	246.1004	[M‐H]^−^	245.0936	2.28	DZTHIGRZJZPRDV‐GFCCVEGCSA‐N
16	6.63	Apigenin‐6,8‐*C*‐di‐pentoside isomer	C25H26O13	534.1373	[M‐H]^−^	533.1298	0.45	‐
17	7.03	Chrysin 6‐*C*‐glucoside 8‐*C*‐arabinoside	C26H28O13	548.1530	[M‐H]^−^	547.1455	0.38	ZGVGUTOTMNVHSX‐UHFFFAOYSA‐N
18	7.11	Apigenin 7‐*O*‐neohesperidoside (Rhoifolin)	C27H30O14	578.1636	[M‐H]^−^	577.1545	3.01	RPMNUQRUHXIGHK‐UHFFFAOYNA‐N
19	7.48	Azelaic acid	C9H16O4	188.1049	[M‐H]^−^	187.0972	1.96	BDJRBEYXGGNYIS‐UHFFFAOYSA‐N
20	7.57	UNKN	C12H20O5	244.1310	[M‐H]^−^	243.1236	0.76	‐
21	8.16	UNKN	C12H20O5	244.1310	[M‐H]^−^	243.1236	0.76	‐

*Note:* RT, retention time. Mass error expressed as parts per million (ppm). UNKN, unknown compound tentatively characterized based on accurate mass. Compound identification was based on accurate mass, isotopic pattern, fragmentation data (MS^E^) and comparison with literature and databases.

### Phytotoxicity in pre‐emergence conditions

In treatments where seed germination was completely inhibited, germination percentage was equal to 0, whereas early growth indices (CVG, MGT, SVI, root and shoot length) could not be calculated and are therefore reported as not determined.

### Impact of *Z. officinale* powder on seed germination and growth of receiver species

**Figure 2:**
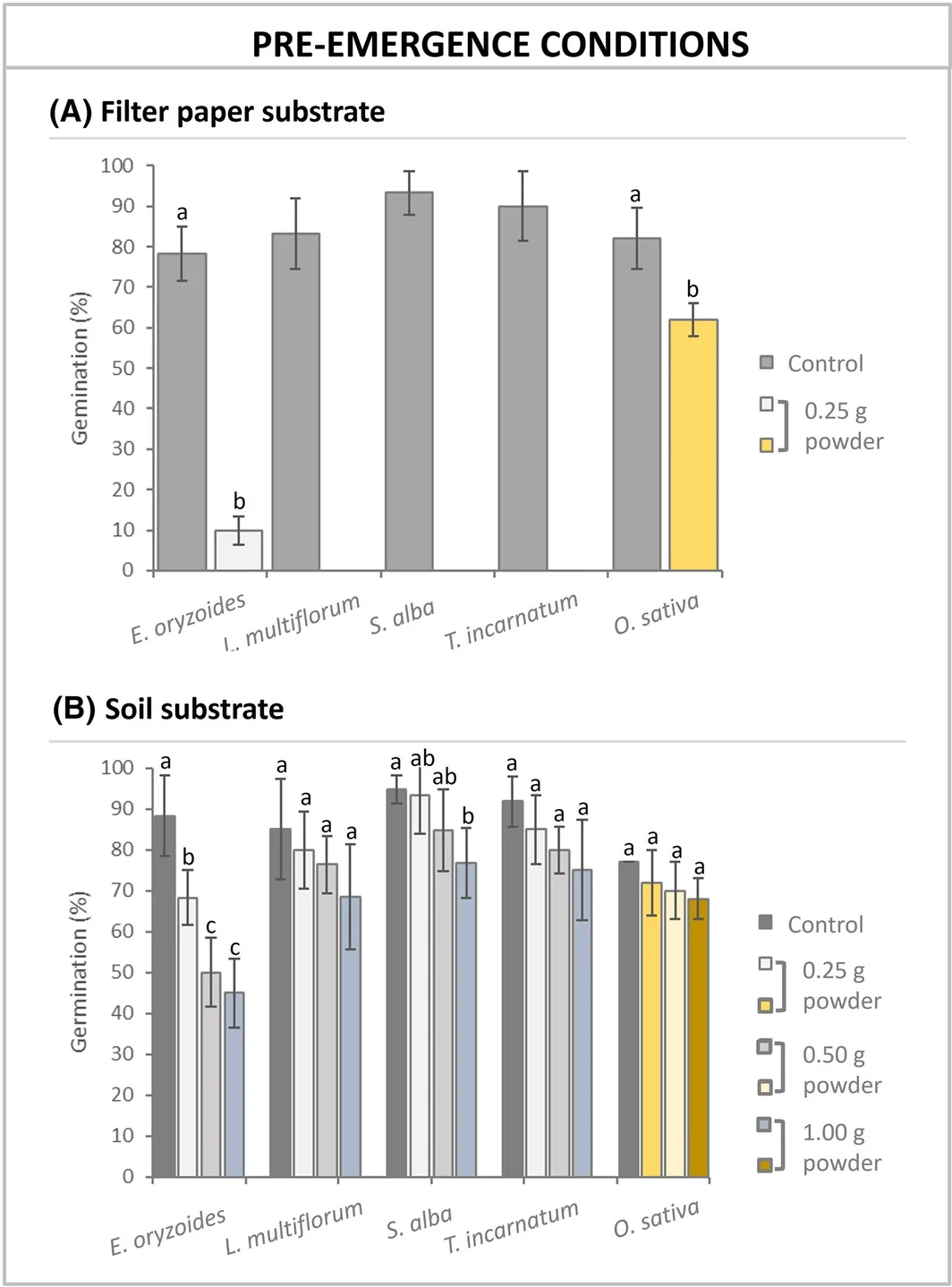
Effect of *Zingiber officinale* leaf powder on seed germination of weed species and *Oryza sativa* under pre‐emergence conditions on (A) filter paper and (B) soil substrates. Leaf powder was applied at 0.25 g per Petri dish on filter paper and at 0.25, 0.50 and 1.00 g per Petri dish on soil. Different letters indicate significant differences among treatments (*P* ≤ 0.05).

On filter paper (Fig. 2(A) and Table 5), *Z. officinale* leaf powder completely inhibited germination of *L. multiflorum*, *S. alba* and *T. incarnatum* at 0.25 g. In *E. oryzoides*, germination decreased by 87.2% (*P* ≤ 0.05), CVG by 97.5% (*P* ≤ 0.05) and SVI by 98.5% (*P* ≤ 0.05). Root and shoot length was reduced by 95.1% and 79.9% (*P* ≤ 0.05), respectively, whereas MGT increased by 25% (*P* ≤ 0.05). *Oryza sativa* was less affected, with a smaller reduction in both germination (−24.4%) and growth indices (CVG, −28.5%; SVI, −40.5%) and an increase in MGT of 20.0%. Overall, the effect of the treatment varied significantly among species for all measured parameters (*P* ≤ 0.001).

**Table 5:** Seed germination and early seedling growth parameters of receiver species after treatment with *Zingiber officinale* leaf powder under pre‐emergence conditions on filter paper

Receiver species	Powder (g)	CVG	MGT	SVI	Root (mm)	Shoot (mm)
*Echinochloa oryzoides*	0	71.1 ± 9.1 a	5.2 ± 0.0 a	6054 ± 627 a	49.5 ± 4.1 a	27.9 ± 2.4 a
	0.25	1.8 ± 0.8 b	6.5 ± 0.0 b	87.5 ± 58.8 b	2.4 ± 1.3 b	5.6 ± 2.0 b
*Lolium multiflorum*	0	88.2 ± 12.3	5.0 ± 0.1	8923 ± 1275	57.2 ± 2.3	49.7 ± 4.1
	0.25	ND	ND	ND	ND	ND
*Sinapis alba*	0	134.2 ± 14.0	4.0 ± 0.0	3620 ± 279	25.0 ± 3.4	13.8 ± 1.8
	0.25	ND	ND	ND	ND	ND
*Trifolium incarnatum*	0	131.8 ± 17.8	4.0 ± 0.0	3620 ± 472	32.5 ± 4.1	7.8 ± 0.6
	0.25	ND	ND	ND	ND	ND
*Oryza sativa*	0	45.2 ± 2.1 a	5.0 ± 0.1 a	3895 ± 431 a	32.4 ± 1.2 a	15.1 ± 0.8 a
	0.25	32.3 ± 2.6 b	6.0 ± 0.2 b	2319 ± 221 b	23.6 ± 0.5 b	11.8 ± 0.3 b

*Note:* Values are the mean ± SD (*n* = 3). Different lowercase letters within the same column and species indicate significant differences between treatments (independent‐samples *t*‐test, *P* ≤ 0.05). CVG, coefficient of velocity of germination; MGT, mean germination time; SVI, seedling vigor index. ND, not determined as a result of complete inhibition of germination. A significant species × treatment interaction was detected for all parameters (two‐way ANOVA, *P* ≤ 0.001).

On the soil substrate (Fig. 2(B) and Table 6), the effects were generally less pronounced. In *E. oryzoides*, germination declined by 22.7–49.1% compared to the control, CVG and shoot length decreased by up to 56.3% and 24.7%, respectively, and MGT increased by 15.2% (all *P* ≤ 0.05).

**Table 6:** Seed germination and early seedling growth parameters of receiver species after treatment with *Zingiber officinale* leaf powder under pre‐emergence conditions on topsoil

Receiver species	Powder (g)	CVG	MGT	Shoot (mm)
*Echinochloa oryzoides*	0	35.4 ± 8.8 a	5.3 ± 0.1 a	26.7 ± 1.4 a
	0.25	27.9 ± 9.7 ab	5.5 ± 0.2 a	26.5 ± 0.7 a
	0.50	22.0 ± 6.1 b	5.7 ± 0.2 ab	23.5 ± 1.5 b
	1.00	15.5 ± 3.8 c	6.1 ± 0.4 b	20.1 ± 2.0 c
*Lolium multiflorum*	0	85.0 ± 3.3 a	4.9 ± 0.2 a	82.7 ± 8.4 a
	0.25	83.5 ± 4.0 a	5.1 ± 0.1 a	79.2 ± 2.9 ab
	0.50	78.1 ± 7.1 a	5.1 ± 0.1 a	73.1 ± 2.8 ab
	1.00	61.0 ± 17.0 a	5.2 ± 0.2 a	68.4 ± 4.0 b
*Sinapis alba*	0	115.4 ± 7.5 a	4.6 ± 0.1 a	33.6 ± 3.0 a
	0.25	109.2 ± 4.2 a	4.7 ± 0.1 ab	33.0 ± 0.7 a
	0.50	84.2 ± 6.7 b	4.9 ± 0.2 b	32.5 ± 2.5 a
	1.00	55.7 ± 7.2 c	5.4 ± 0.2 c	29.8 ± 4.5 a
*Trifolium incarnatum*	0	103.5 ± 5.4 a	4.6 ± 0.1 a	16.2 ± 0.8 a
	0.25	103.2 ± 7.9 a	4.6 ± 0.0 a	15.0 ± 0.7 a
	0.50	84.4 ± 7.1 ab	4.7 ± 0.1 a	14.8 ± 0.9 a
	1.00	63.8 ± 6.8 b	5.3 ± 0.1 b	10.6 ± 2.1 b
*Oryza sativa*	0	26.5 ± 3.0 a	4.2 ± 0.1 a	21.2 ± 1.9 a
	0.25	25.8 ± 2.9 a	4.3 ± 0.1 a	20.4 ± 2.1 a
	0.50	25.0 ± 2.7 ab	4.4 ± 0.1 ab	18.0 ± 0.7 ab
	1.00	24.6 ± 2.5 ab	4.4 ± 0.2 ab	18.1 ± 1.1 ab

*Note:* Values are the mean ± SD (*n* = 3). Different lowercase letters within the same column and species indicate significant differences among treatments (one‐way ANOVA followed by Tukey's test, *P* ≤ 0.05). CVG, coefficient of velocity of germination; MGT, mean germination time. A significant species × treatment interaction was detected for MGT (two‐way ANOVA, *P* ≤ 0.05).

In *L. multiflorum*, germination, CVG and MGT remained unchanged, but shoot length decreased by up to 17.4% at the highest dose (*P* ≤ 0.05). In *S. alba*, germination was reduced by 19.0% (*P* ≤ 0.05) and CVG was reduced by by 51.7% (*P* ≤ 0.001), whereas MGT increased by 17.4% (*P* ≤ 0.001), with no significant changes in shoot length. In *T. incarnatum*, germination was unaffected, but CVG decreased by 38.3% (*P* ≤ 0.01), MGT increased by 15.2% (*P* ≤ 0.001) and shoot length decreased by 34.5% (*P* ≤ 0.001). *Oryza sativa* germination was only slightly reduced (6.5–11.7%) as were the corresponding indices (*P* > 0.05). The response to the treatment differed among species for MGT (*P* ≤ 0.05), whereas no species‐dependent differences were detected for CVG, germination or shoot length.

### *Z. officinale* extract impact on seed germination and growth of the receiver species

**Figure 3:**
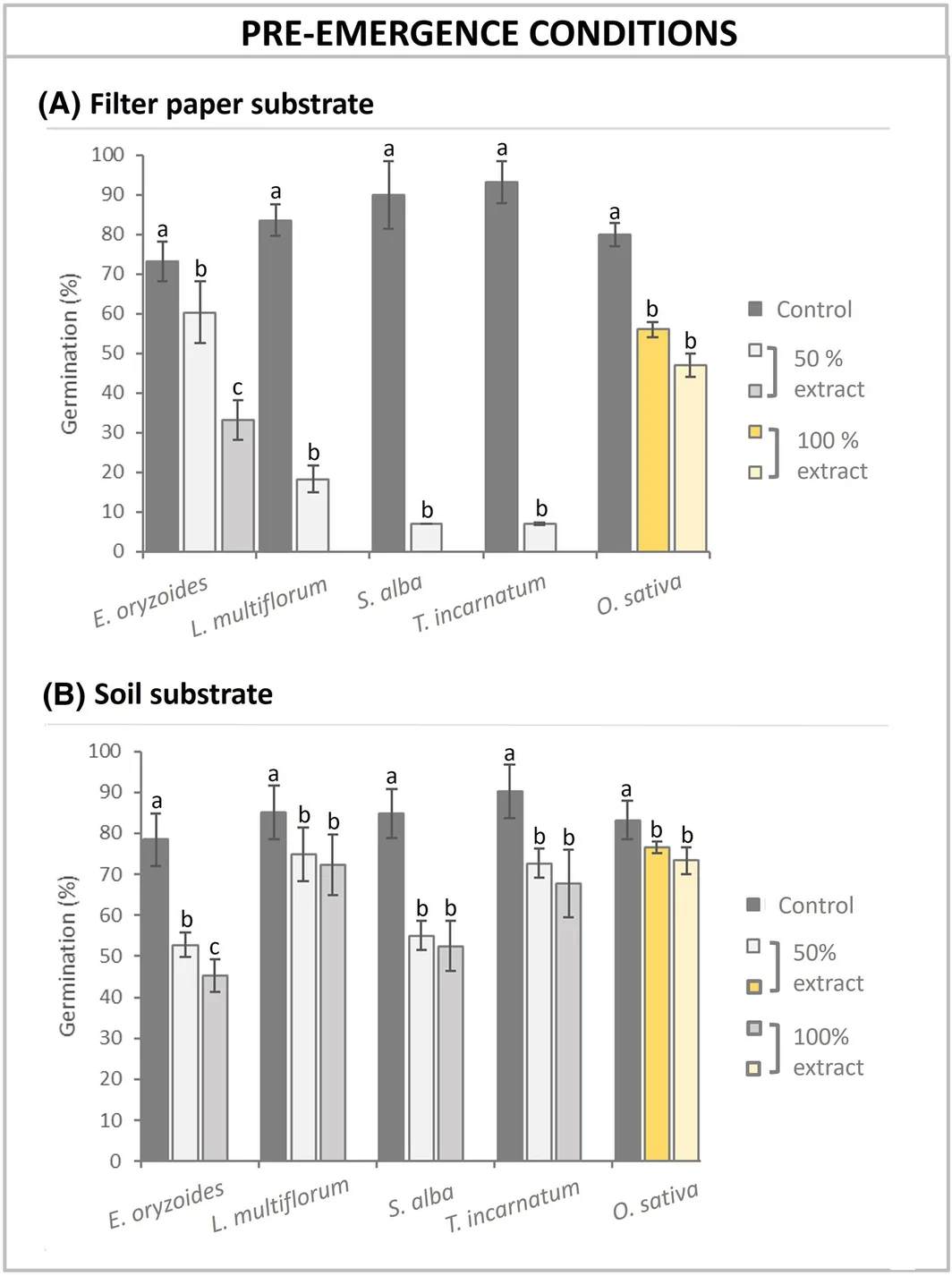
Effect of *Zingiber officinale* leaf aqueous extract on seed germination of weed species and *Oryza sativa* under pre‐emergence conditions on (A) filter paper and (B) soil substrates. The extract was applied at 50% and 100% concentrations (5 and 15 mL per Petri dish for filter paper and soil substrates, respectively). Different lowercase letters indicate significant differences among treatments (*P* ≤ 0.05).

On filter paper (Fig. 3(A) and Table 7), the extract showed strong inhibitory effects. Germination of *L. multiflorum*, *S. alba* and *T. incarnatum* was completely suppressed at 100% concentration. At 50%, germination was already strongly reduced: in *L. multiflorum*, in *S. alba* and in *T. incarnatum* (all *P* ≤ 0.001). In *E. oryzoides*, germination decreased progressively by 17.7% at 50% and 54.6% at 100% (*P* ≤ 0.001), with corresponding reductions in CVG (−41.8% and − 79.3%), SVI (−75.2% and − 93.3%), root length (−90.8% and 97.3%) and shoot length (−29.8% and − 62.5%). MGT increased by 5.5% at 50% and by 11.0% at 100% (*P* ≤ 0.001). *Oryza sativa* germination was less affected (30–41.2%), albeit significantly (*P* ≤ 0.001), with similar effects on growth indices (*P* ≤ 0.001). The effect of the treatment varied significantly among species for all measured parameters (*P* ≤ 0.001).

**Table 7:** Seed germination and early seedling growth parameters of receiver species after treatment with *Zingiber officinale* aqueous extract under pre‐emergence conditions on filter paper

Receiver species	Extract concentration (%)	CVG	MGT	SVI	Root (mm)	Shoot (mm)
*Echinochloa oryzoides*	0	56.7 ± 2.3 a	5.5 ± 0.1 a	4464 ± 422 a	40.2 ± 2.5 a	20.8 ± 1.7 a
	50	33.0 ± 6.7 b	5.8 ± 0.1 b	1108 ± 301 b	3.7 ± 3.2 b	14.6 ± 0.8 b
	100	11.7 ± 2.2 c	6.1 ± 0.1 c	296 ± 62 c	1.1 ± 0.1 b	7.8 ± 0.9 c
*Lolium multiflorum*	0	92.8 ± 4.4 a	4.8 ± 0.1 a	8769 ± 295 a	55.4 ± 1.4 a	49.7 ± 2.5 a
	50	7.4 ± 3.3 b	5.9 ± 0.1 b	493 ± 91 b	4.1 ± 2.7 b	23.1 ± 2.5 b
	100	ND	ND	ND	ND	ND
*Sinapis alba*	0	129.6 ± 16.2 a	4.1 ± 0.1 a	3488 ± 180 a	23.3 ± 1.4 a	15.6 ± 0.7 a
	50	6.0 ± 0.5 b	4.1 ± 0.3 a	75 ± 12 b	3.3 ± 0.5 b	7.5 ± 1.3 b
	100	ND	ND	ND	ND	ND
*Trifolium incarnatum*	0	137.9 ± 11.3 a	4.0 ± 0.0 a	3131 ± 91 a	26.4 ± 1.1 a	7.2 ± 1.0 a
	50	5.8 ± 0.6 b	4.3 ± 0.3 b	54 ± 16 b	4.5 ± 0.6 b	3.3 ± 1.9 b
	100	ND	ND	ND	ND	ND
*Oryza sativa*	0	37.2 ± 3.1 a	6.0 ± 0.3 a	4312 ± 521 a	36.7 ± 3.3 a	17.2 ± 0.9 a
	50	26.0 ± 2.2 b	6.4 ± 0.1 b	2486 ± 325 b	31.2 ± 2.5 b	13.2 ± 1.1 b
	100	21.4 ± 1.6 c	6.6 ± 0.2 c	1866 ± 231 c	27.8 ± 1.2 c	11.9 ± 1.0 c

*Note:* Values are the mean ± SD (*n* = 3). Different lowercase letters within the same column and species indicate significant differences among treatments (one‐way ANOVA followed by Tukey's test, *P* ≤ 0.05). CVG, coefficient of velocity of germination; MGT, mean germination time; SVI, seedling vigor index. ND, not determined as a result of complete inhibition of germination. A significant species × treatment interaction was detected for all parameters (two‐way ANOVA, *P* ≤ 0.001).

On soil (Fig. 3(B) and Table 8), the extract exerted weaker but still significant effects across all species. Germination percentages fell progressively, with reductions of 22.7–49.0% in *E. oryzoides* (*P* ≤ 0.05), 5.9–19.4% in *L. multiflorum* (*P* ≤ 0.05), 1.6–18.9% in *S. alba* (*P* ≤ 0.05), 7.4–18.4% in *T. incarnatum* (*P* ≤ 0.05) and 6.5–11.7% in *O. sativa* (*P* ≤ 0.05). Concomitantly, CVG decreased significantly in all species (*P* ≤ 0.05), with reductions ranging from 15.3% to 50.2%. MGT, although less affected, showed slight but significant increases in most species (up to +12.8%, *p* ≤ 0.01), except in *T. incarnatum*, where it remained unchanged (*P* > 0.05). Shoot length was also consistently reduced, by 14.6–32.5% at 100% extract (*P* ≤ 0.01).

**Table 8:** Seed germination and early seedling growth parameters of receiver species after treatment with *Zingiber officinale* aqueous extract under pre‐emergence conditions on topsoil

Receiver species	Extract concentration (%)	CVG	MGT	Shoot (mm)
*Echinochloa oryzoides*	0	23.3 ± 5.0 a	6.3 ± 0.2 a	28.9 ± 2.8 a
	50	17.5 ± 2.0 b	6.7 ± 0.2 b	22.0 ± 1.5 b
	100	13.5 ± 1.8 c	7.0 ± 0.2 b	19.5 ± 1.2 b
*Lolium multiflorum*	0	79.0 ± 6.7 a	5.1 ± 0.1 a	79.6 ± 1.1 a
	50	71.0 ± 5.5 ab	5.3 ± 0.1 b	70.5 ± 2.3 b
	100	65.5 ± 6.6 b	5.4 ± 0.1 b	68.0 ± 3.1 b
*Sinapis alba*	0	99.3 ± 10.5 a	4.7 ± 0.1 a	37.1 ± 2.0 a
	50	62.0 ± 6.5 b	5.1 ± 0.1 b	30.5 ± 1.6 b
	100	49.5 ± 5.0 c	5.3 ± 0.1 c	28.0 ± 2.0 b
*Trifolium incarnatum*	0	110.4 ± 6.7 a	4.6 ± 0.0 a	28.6 ± 2.1 a
	50	93.5 ± 11.1 b	4.7 ± 0.1 a	22.5 ± 1.2 b
	100	91.5 ± 9.7 b	4.7 ± 0.2 a	20.5 ± 0.9 b
*Oryza sativa*	0	32.0 ± 2.0 a	6.0 ± 0.2 a	32.0 ± 2.1 a
	50	28.0 ± 2.5 ab	6.3 ± 0.3 ab	28.0 ± 2.0 ab
	100	23.8 ± 2.3 b	6.5 ± 0.2 b	25.1 ± 1.8 b

*Note:* Values are the mean ± SD (*n* = 3). Different lowercase letters within the same column and species indicate significant differences among treatments (one‐way ANOVA followed by Tukey's test, *P* ≤ 0.05). CVG, coefficient of velocity of germination; MGT, mean germination time. A significant species × treatment interaction was detected for CVG (two‐way ANOVA, *P* ≤ 0.001).

### Phytotoxicity in post‐emergence conditions

Significant differences in phytotoxic responses to *Z. officinale* aqueous extract were observed among the five tested receiver species (Table 9). The percentage of affected plants ranged from 19% in *L. multiflorum* to complete plant involvement (100%) in *T. incarnatum* and *E. oryzoides*. A similar trend was recorded for affected leaves, with values between 11% in *L. multiflorum* and 98% in *T. incarnatum*. Cumulative phytotoxicity also varied significantly among species, ranging from 20% in *O. sativa* to very high values in *S. alba* (96%) and *T. incarnatum* (98%), with intermediate responses observed in *L. multiflorum* (75%) and *E. oryzoides* (81%). Consistent with pre‐emergence results, *O. sativa* was the least sensitive species, showing limited foliar injury compared to the others.

**Table 9:** Phytotoxic effects observed in receiver species after foliar application of *Zingiber officinale* aqueous extract under post‐emergence conditions

Receiver species	Affected plants (%)	Affected leaves (%)	Cumulative phytotoxicity (%)
*Echinochloa oryzoides*	100 ± 3	95 ± 7	81 ± 4
*Lolium multiflorum*	19 ± 1	11 ± 1	75 ± 5
*Sinapis alba*	86 ± 6	78 ± 5	96 ± 6
*Trifolium incarnatum*	100 ± 6	98 ± 6	98 ± 5
*Oryza sativa*	23 ± 2	18 ± 1	20 ± 1

*Note:* Values are the mean ± SD (*n* = 6). Different lowercase letters within the same column indicate significant differences among species (one‐way ANOVA followed by Tukey's test, *P* ≤ 0.05). Cumulative phytotoxicity was calculated using a semi‐quantitative visual injury scale; symptom classes are described in Table 1.

Qualitative assessment revealed marked differences in the severity and distribution of lesions among species. In *E. oryzoides*, lesions were mostly moderate to strong, predominantly distributed within classes III and V–VII. *Lolium multiflorum* displayed sparse lesions, primarily belonging to classes V and VI, with overall mild symptoms. *Sinapis alba* exhibited a mixed response pattern, with higher severity lesions in classes II–IV and X, indicating both moderate and severe damage. *Trifolium incarnatum* suffered severe injury almost exclusively in class X. By contrast, *Oryza sativa* showed only mild symptoms, limited to classes I—V, with no lesions in higher severity classes, confirming its relative tolerance to the extract.

## DISCUSSION

The present study provides screening‐level evidence of the phytotoxic activity of *Z. officinale* leaf‐derived preparations under controlled pre‐ and post‐emergence conditions. By integrating phytotoxicity testing with complementary chemical analyses, it extends existing bioassay‐based observations through a more comprehensive interpretation of the observed biological responses and of the contribution of both volatile and water‐soluble constituents to the detected activity.

A comparison between leaf powder and aqueous extracts highlighted distinct but complementary patterns of phytotoxicity, reflecting differences in chemical composition, bioavailability and interaction with the substrate. Under inert conditions, both matrices expressed their intrinsic phytotoxic potential, whereas attenuation of effects was consistently observed in soil‐based assays.

This behavior is in line with previous studies showing that soil strongly modulates allelopathic responses through adsorption processes, chemical transformation and microbial activity, resulting in an ecologically realistic reduction of biological effects.^34^, ^35^ Such attenuation aligns with environmentally relevant exposure scenarios and does not imply reduced agronomic relevance.

The lower sensitivity observed for *O. sativa* compared with the tested weed species suggests a degree of differential response that may be relevant from a practical perspective. Such variability may reflect species‐specific physiological and biochemical traits, including differences in seed characteristics, uptake efficiency, metabolic detoxification capacity and sensitivity to allelochemicals.^36^ Differences in growth strategy and phylogenetic background between monocotyledonous and dicotyledonous receiver species may also contribute to the observed response patterns. The inclusion of species differing in ecological behavior, physiological traits and agronomic relevance allowed a preliminary assessment of selectivity patterns under controlled conditions.^37^ In particular, the inclusion of weeds for which herbicide‐resistant biotypes have been reported^6^ increases the relevance of exploring plant‐derived phytotoxic materials as complementary tools within integrated and sustainable weed‐management strategies. Although this selectivity was assessed under controlled conditions, crop‐weed response patterns were reported for plant‐derived residues and extracts, supporting their potential use as low‐impact tools rather than as stand‐alone herbicides.^34^, ^38^

Under pre‐emergence conditions on filter paper, root elongation was generally more strongly inhibited than shoot development, particularly in the most sensitive weed species. This pattern is consistent with previous reports indicating that root tissues are especially susceptible to allelochemical exposure during early seedling establishment because direct contact with phytotoxic compounds occurs during imbibition and early root growth.^34^, ^39^ Root sensitivity to allelochemicals has been associated with alterations in membrane functionality, ion uptake, oxidative balance and cell division processes, ultimately affecting root elongation more markedly than shoot growth.^40^, ^41^

Chemical characterization of *Z. officinale* leaves allowed the identification of constituents potentially contributing to the inhibitory activity observed. Among the volatile compounds, *β*‐caryophyllene emerged as the predominant constituent and was associated with phytotoxic effects in plants, mainly linked to membrane perturbation, oxidative stress induction and interference with photosynthesis‐related processes.^42^, ^43^, ^44^ For other identified sesquiterpenes, such as *γ*‐muurolene and *α*‐curcumene, their common occurrence in allelopathic volatile blends likely reflects the complexity of sesquiterpene‐rich phytochemical profiles, although additive or synergistic contribution to the observed phytotoxic effects cannot be excluded.^45^, ^46^, ^47^, ^48^

Among the monoterpenes, *β*‐pinene, the second most abundant component detected in the aqueous extract, has been widely reported as an allelochemical with documented effects on chlorophyll content and membrane integrity in several crop and weed species. Its presence supports the involvement of a complex volatile mixture rather than single‐compound effects.^49^, ^50^ The non‐volatile fraction further supports a multi‐compound mode of action. UPLC‐HR‐MS and NMR analyses revealed a prevalence of *C*‐glycosylated flavones, mainly apigenin derivatives, including vicenin‐type and related *C*‐glycosides. Flavone *C*‐glycosides have been associated with allelopathic activity, particularly through inhibition of cell division, alteration of auxin transport and interference with redox homeostasis during early seedling development.^51^ Their high abundance in the aqueous extract indicates that water‐soluble phenolics contribute to the phytotoxic responses under both pre‐ and post‐emergence conditions.

The integration of phytotoxicity assays with metabolomic profiling suggests that the observed biological responses are likely associated with a multi‐target mode of action involving both volatile and water‐soluble constituents. Sesquiterpenes and monoterpenes identified in the volatile fraction have been associated with membrane destabilization, reactive oxygen species accumulation, and impairment of photosynthetic processes,^42^, ^43^, ^44^, ^49^, ^50^ whereas flavone *C*‐glycosides may contribute through interference with cell division, auxin balance and redox homeostasis during early seedling development.^51^ The combined occurrence of these metabolites is therefore consistent with the inhibitory effects observed on germination and seedling growth under both pre‐ and post‐emergence conditions. This integrated chemical profile supports the hypothesis that phytotoxicity results from the combined action of multiple metabolites rather than from a single dominant compound.

The coexistence of volatile terpenoids and non‐volatile flavonoids suggests that *Z. officinale* leaves may operate through complementary mechanisms, with volatile components providing rapid effects and water‐soluble phenolics contributing to more persistent activity. This chemical diversity reflects naturally occurring allelopathic systems and is consistent with sustainable weed‐management strategies based on multi‐target, low‐input approaches rather than direct replacement of synthetic herbicides.^39^, ^52^

## CONCLUSIONS

The present study demonstrates that *Z. officinale* leaf biomass contains bioactive constituents capable of affecting seed germination and early seedling development of several weed species under controlled conditions, at the same time as exhibiting lower sensitivity in *O. sativa*. By integrating biological assays with complementary chemical profiling, the present work highlights the potential relevance of aerial *Z. officinale* biomass as a biologically active agricultural by‐product within circular‐economy‐oriented agricultural systems.

Because the observed effects derive from lab‐scale assays, further research is required to optimize extraction and formulation strategies, assess crop safety across different soils and climatic contexts, and verify efficacy under agronomically realistic conditions.

## FUNDING INFORMATION

This work was supported by the European Agricultural Fund for Rural Development (FEASR) through the Rural Development Programme (PSR) 2014–2020 of Regione Lombardia, within the framework of the ‘PRECISION WEED’ project. The funders had no role in study design, data collection, analysis and interpretation, writing of the manuscript, or decision to submit the article for publication.

## CONFLICTS OF INTEREST

The authors declare that they have no conflicts of interest.

## AUTHOR CONTRIBUTIONS

SV and MI were responsible for conceptualization, data curation, methodology, project administration, supervision and reviewing and editing. SV, OI, SF, AP, SG, CA and MI were responsible for formal analysis. SV, OI, SF and MI were responsible for investigations. SV, AP, SG, VV, CA and MI were responsible for validation. SV, OI, AP, SG, CA and MI were responsible for visualization. SV, SF, AP, SG, VV and CA were responsible for writing the original draft. AP, SG, CA and MI were responsible for resources.

## Data Availability

The data that support the findings of this study are available from the corresponding author upon reasonable request.
